# Predictive Models for Early Infection Detection in Nursing Home Residents: Evaluation of Imputation Techniques and Complementary Data Sources

**DOI:** 10.3390/healthcare14020166

**Published:** 2026-01-08

**Authors:** Melisa Granda, María Santamera-Lastras, Alberto Garcés-Jiménez, Francisco Javier Bueno-Guillén, Diego María Rodríguez-Puyol, José Manuel Gómez-Pulido

**Affiliations:** 1 Department of Medicine and Medical Specialties, Universidad de Alcalá (UAH), 28805 Alcalá de Henares, Madrid, Spain; maria.santamera@uah.es (M.S.-L.); drodriguez.hupa@gmail.com (D.M.R.-P.); 2 Health Computing and Intelligent Systems Research Group (HCIS), Universidad de Alcalá (UAH), 28805 Alcalá de Henares, Madrid, Spain; alberto.garces@uah.es (A.G.-J.); fjavier.bueno@uah.es (F.J.B.-G.); jose.gomez@uah.es (J.M.G.-P.); 3 Ramón y Cajal Institute for Health Research (IRYCIS), 28034 Madrid, Spain; 4 Department of Computer Science, Universidad de Alcalá (UAH), 28805 Madrid, Spain

**Keywords:** infectious diseases, physiological data, imputation techniques, machine learning, early diagnosis, elderly people, interpretability

## Abstract

**Background:** Aging in Western societies poses a growing challenge, placing increasing pressure on healthcare costs. Early identification of infections in elderly nursing home residents is crucial to reduce complications, mortality, and the burden on emergency departments. **Methods:** We performed a comparative analysis of machine learning models using XGBoost classifiers for infection detection, addressing incomplete daily physiological measurements (Heart Rate, Oxygen Saturation, Body Temperature, and Electrodermal Activity) through strict imputation protocols. We evaluated three model variants—Basic (clinical only), Air Pollution-added, and Social Media-integrated—while incorporating a novel Basal Module to personalize physiological baselines for each resident. **Results:** Results from the binary model indicate that physiological data provides a necessary baseline for immediate screening. Notably, social media integration emerged as a powerful forecasting tool, extending the predictive horizon to a 6-day lead time with an F1-score of 0.97. Complementarily, air pollution data ensured robust immediate detection (“nowcasting”). In the multiclass scenario, external data resolved the “semantic gap” of vital signs, improving sensitivity for specific infections (e.g., acute respiratory and urinary tract infections) to over 90%. **Conclusions:** These findings highlight that the strategic integration of environmental and digital signals transforms the system from a reactive monitor into a proactive early warning tool for long-term care facilities.

## 1. Introduction

**Figure 1:**
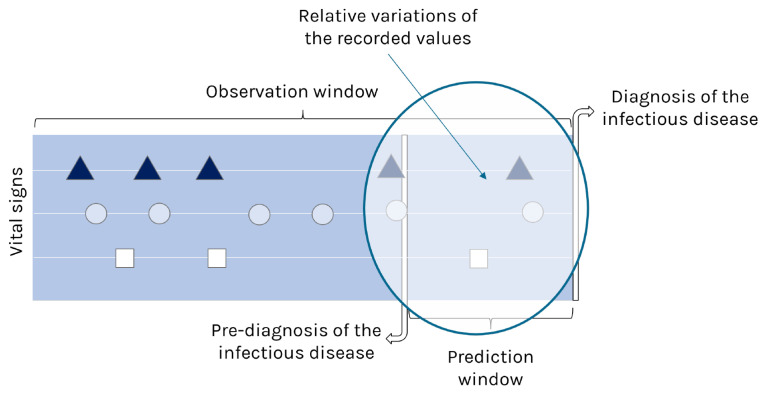
Timeline of early infectious disease prediction. The diagram illustrates the stream of vital signs (represented by geometric shapes) over time. The “Pre-diagnosis” vertical line divides the data into an Observation Window (actual historical data) and a Prediction Window (future projection). The circle highlights the relative variations estimated by the model before the formal clinical diagnosis occurs (far right), allowing for early intervention.

The rapid aging of the population places increasing pressure on healthcare systems, making early disease detection critical for reducing dependency and hospital costs [1]. In geriatric care, a major challenge is the high rate of preventable hospitalizations due to the late detection of infectious decompensations in frail elderly patients. Standard monitoring often relies on “one-size-fits-all” vital sign thresholds that fail to account for the high inter-subject variability of this population. To address this, we propose a personalized telemonitoring system that shifts away from static alerts. Our solution utilizes a Basal Module to detect relative physiological changes per patient and integrates environmental context to identify “Red Flags” before severe clinical deterioration occurs. The biological premise of this approach lies in the incubation period: before overt symptoms manifest, pathogen proliferation triggers subtle physiological changes in vital signs [2]. We hypothesize that detecting these early variations allows for intervention during the “observation window,” well before the clinical onset of the disease. As illustrated in Figure 1, the timeline is divided into two distinct phases: the observation window, where historical baselines are established, and the prediction window, which captures the relative variations enabling an early, data-driven pre-diagnosis.

This study focuses on the early detection of Acute Respiratory Infections (ARI) and Urinary Tract Infections (UTI), the predominant causes of morbidity in geriatric populations [3,4], alongside a third class labeled “Others”—a heterogeneous category defined by exclusion for clinical presentations that require care but do not meet specific ARI/UTI criteria, such as Skin and Soft Tissue Infections (SSTI).

Building upon the SPIDEP project [5], this work addresses a fundamental obstacle in diverse data integration: data incompleteness. The impact of missingness on model performance depends on its underlying mechanism, traditionally classified as Missing Completely at Random (MCAR), Missing at Random (MAR), or Missing Not at Random (MNAR) [6,7,8]. For this study, we hypothesize that the data follows an MAR mechanism, as gaps in this specific nursing home setting are largely driven by operational factors—such as staff shifts, device availability, and variations in daily routines—rather than the patient’s health status itself. Under this MAR assumption, we opted for Multiple Imputation by Chained Equations (MICE) and k-Nearest Neighbors (kNN) as our primary strategies. Unlike simple mean approaches that often distort data distributions [9], MICE and kNN are designed to preserve the multivariate covariance structure and complex variability [10], ensuring that imputed values remain biologically consistent, such as maintaining the correlation between fever and tachycardia, without fabricating unrealistic patterns [11,12].

Beyond data completeness, accurately capturing the environmental context is essential for robust disease modeling. Meteorological variations and air pollution levels are known to modulate immune responses [13], though these effects are often delayed. To address this, we implement a ‘Lagged Model’, shifting exposure data by a predefined window to capture causal relationships rather than spurious correlations [14,15,16]. Complementing these physical indicators, digital epidemiology has emerged as a vital surveillance tool [17,18,19,20]. By leveraging signals from sources like Google Trends, AI-driven systems can detect early outbreak signatures in the community that traditional surveillance might miss [21]. These digital signals serve as proxy indicators for the regional viral load, adjusting the model’s sensitivity during periods of high infection risk [22].

Consequently, this study presents a comprehensive evaluation of machine learning frameworks using an XGBoost Classifier. Our main contributions include identifying the research gap regarding personalized baselines, developing the Basal Module to handle clinical heterogeneity, and demonstrating that integrating pollution and digital context provides incremental sensitivity gains for infection detection, ultimately providing a validated tool to support clinical decision-making in high-demand emergency settings.

To address the existing research gap regarding the lack of personalization in standard geriatric monitoring, this study presents a comprehensive evaluation of machine learning frameworks. Our main contributions include the development of a novel Basal Module to handle clinical heterogeneity through personalized baselines, and the demonstration that integrating air pollution and digital epidemiology provides incremental sensitivity gains for infection detection. The remainder of this article is organized as follows: Section 2 describes the multi-source dataset and the methodological pipeline; Section 3 presents the experimental results comparing the proposed architectures; Section 4 discusses the clinical implications, explainability, limitations of the findings and future directions; and Section 5 offers concluding remarks.

## 2. Materials and Methods

### 2.1. Study Design and Multi-Source Data Integration

This longitudinal study was conducted over a period of 16.5 months in two nursing homes located in Madrid, Spain. The research protocol was approved by the Research Ethics Committee of the Fundación para la Investigación Biomédica del Hospital Universitario Príncipe de Asturias, Spain (favorable advice number OE 21/2016), which waived patient consent requirements given the fully anonymized nature of the dataset. The study focused on a cohort of 60 geriatric residents (average age: 87.6 years), a demographic selected due to their high vulnerability to infectious outbreaks. This monitoring campaign resulted in a total of 5004 valid samples of daily physiological measurements, with further population metrics summarized in Table 1.

Clinical data included labels provided by doctors classifying each daily sample as either “Healthy” or “Infected.” The infections were categorized into ARI, UTI, and “Other Infections” (mainly SSTI), as seen in Table 2. Although the study population consisted of 60 residents, only 43 presented with infections; the total of 152 diagnostic events indicates recurrence, resulting in an average of 3.53 infections per affected resident.

To construct a comprehensive predictive framework, we integrated data from three distinct sources:

- **Physiological Data (Internal Source):** Measurements were collected daily using wearable biosensors recording Body Temperature (TEMP), Electrodermal Activity (EDA), Oxygen Saturation (SpO_2_), and Heart Rate (BPM). Trained medical personnel oversaw collection according to established protocols described in the SPIDEP Project [5]. These were complemented by demographic data (age, sex) and clinical characteristics, specifically the Barthel Index, which measures the patient’s performance in activities of daily living and functional independence.
- **Environmental Data (External Source):** Recognizing the influence of environmental factors on respiratory health, daily meteorological variables (mean temperature, pressure, insolation) and air pollution metrics (e.g., Nitrogen Dioxide (NO_2_), Particulate Matter (PM_10_)) were retrieved from public repositories and temporally matched with physiological data.
- **Digital Epidemiology Data (External Source):** To capture social context and potential early warnings of community transmission, we integrated search volume data from Google Trends. We selected specific keywords in Spanish related to respiratory symptoms (e.g., “flu”, “fever”) as well as urinary tract symptoms (e.g., “cystitis”, “dysuria”) to analyze local search behaviors during the study period. The complete list of Spanish keywords used for the Google Trends data extraction, including the comparison between raw and refined sets, is provided in Supplementary Table S1.

To systematically assess the predictive value of these diverse data streams and optimize the feature space, we designed a comparative experimental framework comprising three progressive modeling scenarios:

- **Basic Model:** Utilizing exclusively demographic data and physiological vital signs (internal source).
- **Air Pollution Model:** Incorporating pollutant variables (internal + environmental sources).
- **Social Media Model:** Integrating digital epidemiology metrics from Google Trends (internal + digital sources).

For each of these scenarios, two distinct feature sets were evaluated: a Full Feature Set, containing all available variables, and a Selected Feature Set, derived from a SHAP (SHapley Additive exPlanations) analysis to identify the most relevant predictors. Furthermore, to evaluate the system’s early warning capabilities, the models were trained and tested across varying temporal windows, modifying the Lead (observation window) and Lag (prediction horizon) parameters to determine the optimal forecasting timeframe.

### 2.2. Data Preprocessing and Missing Data Imputation

Given the significant disparity between classes, we employed random undersampling of the majority class to prevent model bias towards the dominant label.

The distribution of missing data across vital signs is summarized in Table 3, indicating a high prevalence of incomplete records for all variables. These missing values were primarily attributed to two factors: the non-continuous nature of clinical monitoring in residential care settings—where vitals are typically recorded during specific nursing shifts rather than through automated bedside telemetry—and occasional technical disruptions in data transmission from the environmental sensors. Consequently, handling data gaps was a critical step to maintain the temporal continuity required for the Basal Module.

Three imputation strategies were employed:

- Mean Imputation: Missing values were replaced with the average value of each feature.
- kNN: Missing data were estimated using similarity-based predictions from the closest neighbors in the dataset, preserving relationships between variables, and capturing local data patterns.
- MICE: Multiple imputed datasets were generated iteratively to address uncertainty and variability in missing values, enhancing the robustness and reliability of the subsequent analyses.

To ensure physiological plausibility and prevent the fabrication of unrealistic patterns, we enforced a strict maximum imputation window of 4 days. Gaps exceeding this threshold were treated as independent discontinuous sequences and were not imputed. Crucially, to avoid any artificial inflation of performance, no imputed data were used for testing; the evaluation of all models was conducted exclusively on real, observed clinical segments.

### 2.3. Basal Module

To address the challenge of high inter-subject variability in geriatric patients, we developed the Basal Module, a mathematical framework designed to transform absolute physiological values into personalized relative indicators. Rather than comparing a resident’s vital signs against universal clinical thresholds—which often lead to false alerts in frail populations—this module establishes an adaptive baseline for each individual.

For each physiological variable at time t, the module maintains a historical set $H_{T}=\{x_{1},\ldots ,x_{t-1}\}$ representing the resident’s previous basal state. From this distribution, the empirical quartiles ($q_{0.25},\;q_{0.50},q_{0.75}$) are recursively updated. We then compute two core features:

- **Categorical State (**$Q_{t}$**):** A discretized variable taking values in depending on which historical quartile the current observation falls into.
- **Dynamic Variation (**$∆Q_{t}$**):** Defined as the first-order difference $Q_{t}-Q_{t-1}$, capturing sudden shifts in the patient’s physiological state.

This quantization process acts as a non-linear filter that smooths high-frequency noise while preserving significant state transitions. By converting absolute measures (e.g., a heart rate of 100 BPM) into relative quartiles, the model decouples the signal from the specific individual’s baseline. This allows the XGBoost classifier to learn a universal “deviation signature” rather than memorizing patient-specific identities, effectively mitigating the risk of identity leakage.

### 2.4. System Workflow and Decision Architecture

**Figure 2:**
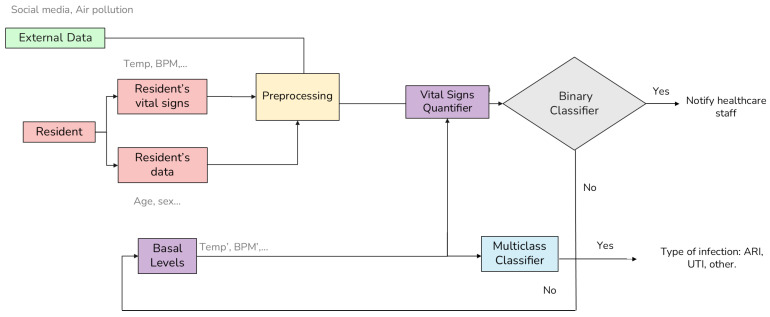
Architecture of the infection prediction system. The diagram illustrates the data flow from external data and resident sensors. The preprocessing stage includes missing data imputation, dataset construction, and random undersampling to address class imbalance. Subsequently, the Vital Signs Quantifier normalizes readings against the patient’s historical Basal Levels. The processed signals (Q,∆Q) feed into a dual-stage decision process: a Binary Classifier for immediate staff notification and a Multiclass Classifier to identify specific infection types (e.g., Acute Respiratory Infections (ARI), Urinary Tract Infections (UTI)). Note the feedback loops (bottom arrows) where non-events update the basal profile to improve future accuracy.

Once the personalized features (Q and ∆Q) are computed by the Basal Module, the overall operational logic of the system integrates them into a multi-stage pipeline. As illustrated in Figure 2, the process begins with the synchronization of multi-domain data: external data, resident-specific information (age, sex, and Barthel Index), and real-time vital signs. These signals are processed through the Basal Module to normalize them into the previously defined individual quartiles.

These refined features are then processed by a dual-classification architecture. First, a Binary Classifier acts as a primary screening tool to determine the presence of an infectious process; if an anomaly is detected, the system triggers a notification to healthcare staff. Simultaneously, a Multiclass Classifier analyzes the signal to categorize the event into specific etiologies: ARI, UTI, or Others (non-specific clinical consultations). This modular design ensures that the system not only alerts to a change in health status but also assists in clinical triage by suggesting the likely nature of the infection.

### 2.5. Temporal Framework: Lead, Lag, and Labeling Strategy

**Figure 3:**
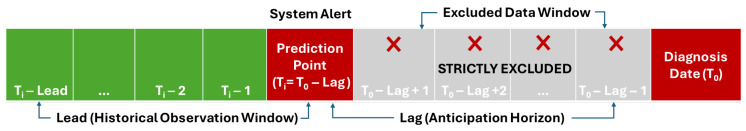
Temporal alignment of infection onset versus clinical diagnosis. The timeline divides the monitoring period into a “Lead” phase (Green, days −15 to −2) representing the pre-infection baseline, and a “Lag” phase (Gray). Note that Day T_i_ marks the biological onset of the infection, while Day T_0_ indicates the later moment of confirmation by the clinician.

To ensure the model captures early physiological signatures and maintains strict predictive integrity, we established a temporal framework based on Lead and Lag variables. As illustrated in Figure 3, the timeline for each case is structured relative to the clinical diagnosis date ($T_{0}$):

1. **Lag (Anticipation Horizon):** The Lag is defined as the number of days prior to that the system aims to anticipate the infection. This creates a “Prediction Point” at $T_{t}=\;T_{0}-\;Lag$. To guarantee that the model does not “peek” into the future, all physiological data occurring between the Prediction Point ($T_{t}$) and the Diagnosis Date ($T_{0}$) are strictly excluded from the feature extraction process. This ensures the model only reacts to early, subtle variations rather than the overt clinical deterioration that immediately precedes diagnosis.
2. **Lead (Historical Observation Window):** The Lead defines the window of historical context used as input for the model, looking backward from the Prediction Point $T_{i}$. The feature vector for a sample is derived exclusively from the interval $\left[T_{i}-\;Lead,\;T_{i}\right]$. By shifting the observation window to end exactly at the Prediction Point, we maintain absolute causal integrity, ensuring that no information from the period $\left[T_{i}+\;1,\;T_{0}\right]$ is encoded in the inputs.

Within these clinically valid ranges, we performed a Grid Search to empirically identify the optimal combination of Lead and Lag that maximized predictive performance. To avoid noisy labels, days with overlapping infections were excluded, and consecutive infections were treated as distinct events only if separated by a washout period of at least 15 days.

### 2.6. Model Training and Evaluation

Two classification frameworks were established to predict infections:

- Binary classification: Differentiating between “healthy” and “infected” states without specifying infection types.
- Multiclass classification: Differentiating specific infection types including “ARI”, “UTI” and “Other Infections”.

For both binary and multiclass classification tasks, we employed the XGBoost algorithm. This choice was motivated by its superior ability to handle non-linear relationships in tabular clinical data and its robustness against class imbalance. To ensure the model’s generalizability and prevent “identity leakage,” we implemented a Stratified Group K-Fold Cross-Validation scheme.

Hyperparameter optimization was conducted via a Randomized Search with 5-Fold Cross-Validation. Unlike standard accuracy, which can be misleading in imbalanced datasets, our optimization objective was to maximize the Matthews Correlation Coefficient (MCC). MCC was selected because it provides a balanced evaluation by considering all four quadrants of the confusion matrix (True Positive (TP), True Negative (TN), False Positive (FP), False Negative (FN)), making it particularly robust for clinical “Red Flag” systems where both sensitivity and specificity are critical. Crucially, this choice aligns with the clinical imperative of this study: in a vulnerable geriatric population, the cost of an FN (missing an active infection) far outweighs that of an FP (a precautionary medical review), necessitating a metric that penalizes missed detections more rigorously than simple accuracy.

### 2.7. Feature Importance and Explainable AI with SHAP

To interpret model predictions, SHapley Additive exPlanations (SHAP) values were computed. SHAP quantifies each feature’s contribution to individual predictions, facilitating interpretability by identifying variables that contribute most significantly to the model outcomes. Specifically, SHAP was employed to assess and visualize the relative importance of vital signs, demographic characteristics, and external variables, highlighting their specific contributions to prediction outcomes. Beyond model interpretability, SHAP values were also used as a criterion for feature selection. A threshold-based strategy was implemented: only features whose mean absolute SHAP value exceeded a predefined threshold were retained for model training. This approach allowed the removal of variables with negligible influence on model outputs, reducing dimensionality while preserving the most informative features. By focusing the model on relevant inputs, this strategy aims to improve generalization, especially in complex multiclass scenarios where irrelevant variables can introduce noise and reduce predictive performance.

## 3. Results

### 3.1. Binary Classification: Early Detection Capacity

The primary objective of the binary classifier was to detect the presence of any infectious process (ARI, UTI, or Other) versus a healthy basal state. Table 4 details the performance of the three modeling scenarios. To ensure the reliability of these results in a clinical setting with intermittent data, the evaluation focuses on the Selected Feature Set (optimized via SHAP) using the mean imputation strategy, which demonstrated superior stability over kNN and MICE approaches.

#### 3.1.1. Impact of Multi-Source Integration

The results reveal that physiological monitoring establishes a strong baseline for detection, but external data sources act as critical signal amplifiers. The Basic Model achieved a sensitivity (recall of the infected class) of 0.84 and a specificity (recall of the healthy class) of 0.73. This suggests that vital signs deviations alone can correctly identify the majority of infectious events but may struggle to filter out all false alarms.

#### 3.1.2. Model Robustness and Specificity

While both external models improved detection rates, the Air Pollution Model offered the most robust balance. It achieved the highest specificity (0.77), outperforming both the Basic Model (0.73) and the Social Media Model (0.72). This suggests that meteorological and pollution variables provide stable contextual markers that help the system rule out false positives.

Conversely, while the Social Media Model matched the high sensitivity of the Air Pollution Model (0.86), it exhibited a slight drop in specificity to 0.72. This decline supports the hypothesis of “social noise” inherent in internet search behaviors, where anxiety-driven searches in the community may precede actual infection trends, leading to a slightly higher rate of false alerts compared to the baseline.

#### 3.1.3. Feature Selection and Efficiency

Crucially, the performance metrics using the Selected Feature Set (variables identified as significant by SHAP) matched or exceeded those of the Full Feature Set. This indicates that the feature selection process successfully filtered out irrelevant variables without compromising diagnostic capability, resulting in a more parsimonious and interpretable model.

### 3.2. Multiclass Diagnosis: Etiological Differentiation

Beyond simple detection, the system was evaluated on its ability to discriminate between specific infection types: ARI (Class 1), UTI (Class 2), and Other Infections (Class 3), versus the Healthy state (Class 0). Table 5 presents the comparative performance metrics for the three modeling scenarios.

#### 3.2.1. The Limitations of Vital Signs Alone

A critical finding is the inability of the Basic Model to perform differential diagnosis. While it successfully detected general anomalies in the binary task, its performance deteriorated significantly in the multiclass setting, with F1-scores dropping below 0.50 for specific infections (e.g., 0.18 for ARI Class and 0.49 for UTI Class). This illustrates that physiological signs are non-specific: hyperthermia or tachycardia are systemic responses common to multiple pathologies, making it impossible for the model to distinguish an ARI from a UTI based solely on internal sensors.

**Figure 4:**
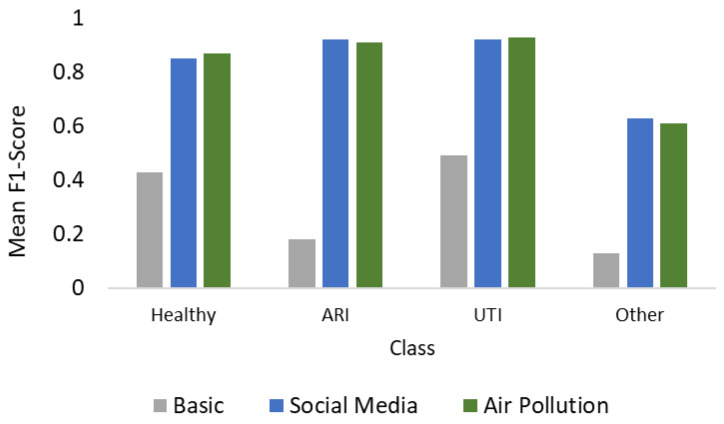
Comparative F1-Score performance across diagnostic classes. The visualization demonstrates the significant performance gap between the Basic Model (gray) and the external data models (blue and green), particularly for ARI and UTI where physiological signs alone failed to provide sufficient discriminative power.

This contrast is visually summarized in Figure 4, where the performance collapse of the Basic Model is evident across all pathological classes. The substantial leap in F1-scores—represented by the colored bars—validates the hypothesis that external contextual layers are required to resolve the ambiguity of raw physiological signals.

#### 3.2.2. Impact of External Data

The integration of external data proved to be the decisive factor for etiological classification. Both the Air Pollution and Social Media models achieved a dramatic performance boost, raising F1-scores to over 0.90 for the primary infection classes (ARI and UTI).

- Social Media Model: Achieved the highest consistency, with a Recall of 0.94 for ARI and 0.97 for UTI. This suggests that community search patterns (e.g., keywords like “dysuria” vs. “cough”) provide the specific semantic labels needed to disambiguate the physiological signal.
- Air Pollution Model: Also demonstrated exceptional performance (Recall > 0.95 for both ARI and UTI), likely leveraging meteorological correlations (e.g., temperature drops associated with respiratory outbreaks).

#### 3.2.3. Challenge of Heterogeneous Classes

Both models struggled more with Class 3 (“Other Infections”), achieving recalls of approximately 0.50. This is expected, as this category includes diverse pathologies (e.g., skin infections) that lack a strong seasonal or digital footprint compared to respiratory or urinary tract infections.

### 3.3. Temporal Analysis: Impact of Lead and Lag

While the previous section evaluated the general consistency of the models through average metrics, this section analyzes the upper performance bound to determine the optimal temporal configuration. To this end, we identified the maximum F1-Score for each anticipation window (Lag), dynamically optimizing the historical data window (Lead) within a range of 2 to 15 days.

#### 3.3.1. Binary Dynamics

**Figure 5:**
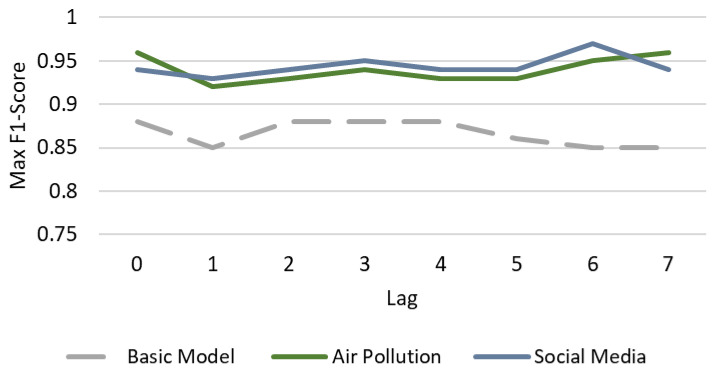
Predictive horizon in binary anomaly detection (Healthy vs. Pathological). Data points represent the maximum F1-Score achieved for each anticipation window (*Lag*) after optimizing the historical data window (*Lead*). The Basic Model (gray) degraded over time, while Social Media (blue) peaked at a 6-day anticipation (F1 0.97), and Air Pollution (green) maintained high stability throughout the week.

Figure 5 presents the system’s performance ceiling in the general classification task (Healthy vs. Pathological). The analysis reveals that the inclusion of external data not only improves accuracy but also extends the prediction lifespan.

The model based exclusively on vital signs showed a stable performance across the temporal window (F1-Score ~0.88). However, it plateaued at this level and failed to reach the high predictive peaks observed in external models.

In contrast, external models maintained performance above 0.90 throughout the week. Notably, the Social Media Model achieved its binary peak with a 6-day anticipation (F1-Score reaches 0.97). This suggests that generalized “digital noise” regarding physical malaise acts as a robust precursor, allowing for alerts on population health anomalies nearly a week prior to clinical saturation.

#### 3.3.2. Multiclass Dynamics

**Figure 6:**
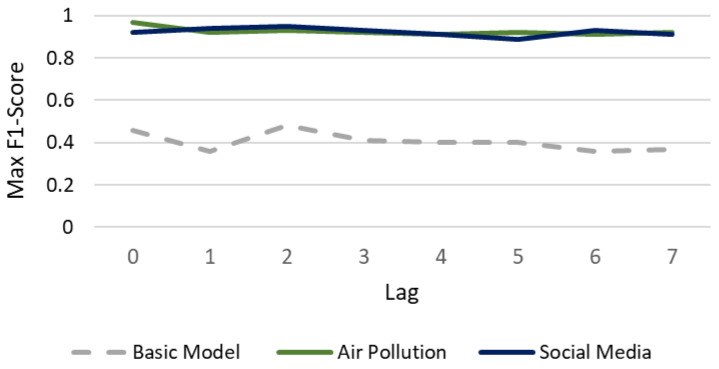
Comparative performance in multiclass etiological diagnosis (ARI vs. UTI vs. Others). Curves display the maximum F1-Score per lag, selected from the optimal Lead configuration (2–15 days). The Basic Model (gray) remains below 0.50 regardless of optimization, illustrating its diagnostic insufficiency. In contrast, Air Pollution (green) maximizes immediate detection (Lag 0, F1 0.97), whereas Social Media (blue) offers an optimal early warning at 2 days (Lag 2, F1 0.95).

The diagnostic challenge increases significantly when distinguishing between specific pathologies (ARI vs. UTI vs. Others), as shown in Figure 6. In this setting, the Basic Model collapsed, failing to exceed an average F1-Score of 0.48 even with optimized temporal windows. This indicates that vital signs alone lack the semantic resolution required for differential diagnosis. Conversely, the external models exhibit a complementary synchronization. The Air Pollution Model achieved its absolute maximum (F1 0.97) in real-time (*Lag* 0), effectively “nowcasting” the immediate physiological impact of air quality. Meanwhile, the Social Media Model optimized its specific diagnostic performance with a 2-day anticipation (F1 0.95), reflecting the behavioral latency between symptom searches and clinical visits.

### 3.4. Model Interpretability and Feature Importance

**Figure 7:**
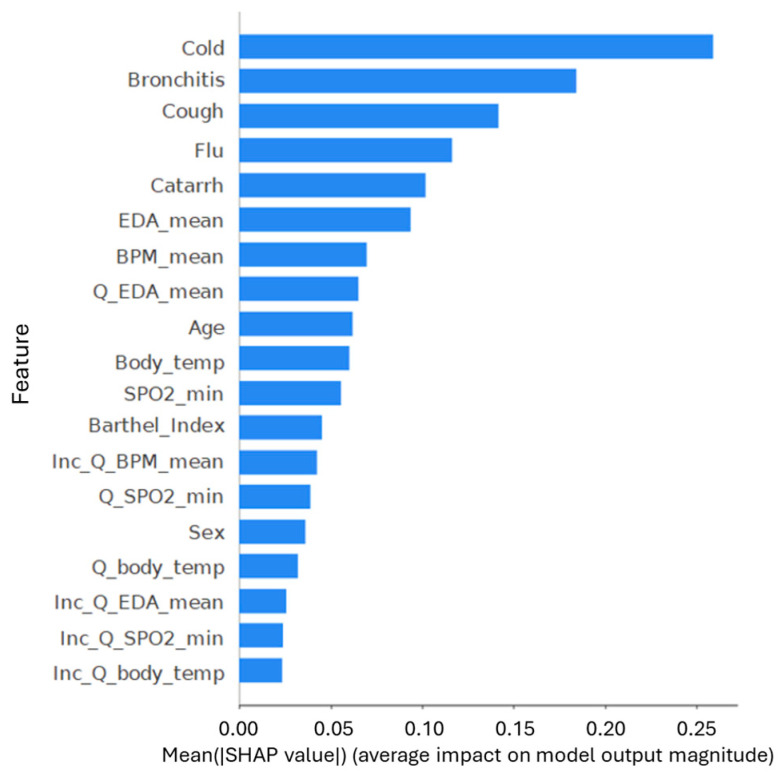
Feature importance ranking based on mean SHAP values. The horizontal bar chart displays the average absolute impact of each feature on the binary model’s output magnitude. The top five features correspond exclusively to respiratory symptom search terms (e.g., Cold, Bronchitis, Cough), which demonstrate a significantly higher predictive contribution than physiological variables (such as EDA_mean or Body_temp).

To evaluate the relevance of specific features within the Social Media-based model and ensure the clinical reliability of its predictions, SHAP values were computed. Figure 7 displays the global importance of each variable for the binary classification scenario. The analysis revealed that the most influential features are terms related to respiratory symptoms—particularly “cold”, “bronchitis”, “cough”, and “flu”. These results indicate a strong relationship between the frequency of symptom-related terms in social discourse and the model’s prediction of general infection risk, effectively validating the use of digital traces as proxies for community health status.

**Figure 8:**
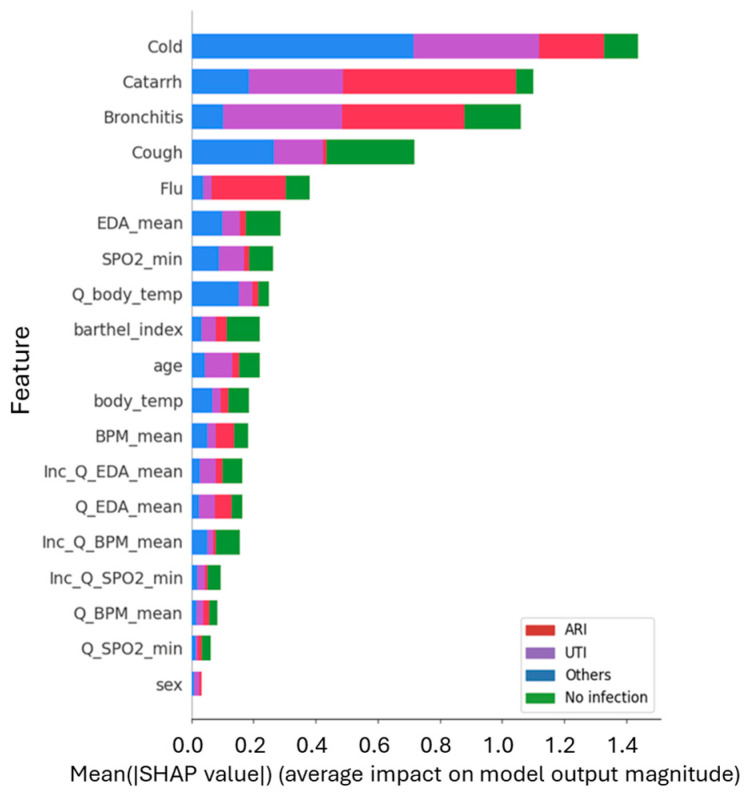
Feature contribution by class for the multiclass diagnosis model. The stacked bar plot illustrates the Mean Absolute SHAP values, identifying which features drive predictions for each specific outcome: ARI (Red), UTI (Purple), Others (Blue), and No Infection (Green). Search terms remain the dominant predictors. Notably, symptoms like Bronchitis and Catarrh show a strong contribution to the ARI classification (red segments), validating the semantic link between the search term and the medical diagnosis.

In the multiclass setting, the decomposition of feature importance across classes is shown in Figure 8. Symptom keywords again dominated the top positions—especially “cold,” “catarrh,” “bronchitis,” and “cough”—but with differentiated influence across the outcome categories, providing the semantic resolution necessary to distinguish between pathologies. Crucially, physiological variables such as EDA mean, SpO2 min, and BPM mean showed moderate but consistent contributions across all classes. This suggests a hierarchical decision process where vital signs establish a baseline of physiological anomaly, while semantic keywords provide the specific context for classification.

**Figure 9:**
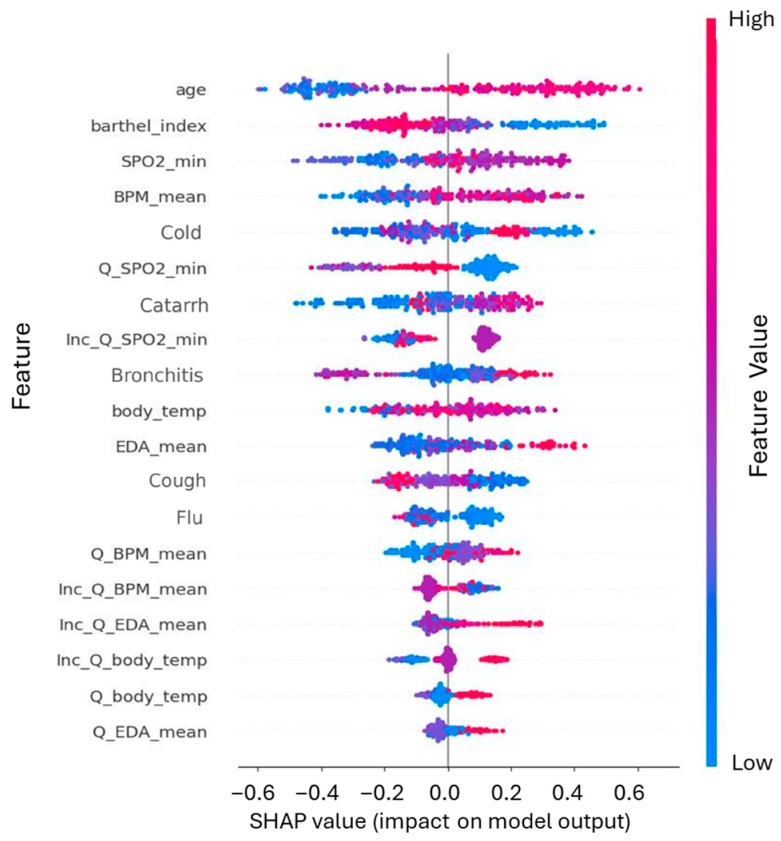
SHAP beeswarm summary plot illustrating feature impact directionality. The plot combines feature importance (*y*-axis ranking) with feature effect. Each dot represents a single instance. The color indicates the feature value (Red = High, Blue = Low), and the horizontal position shows the impact on the model’s output (positive SHAP values indicate higher infection risk). The distribution reveals clear clinical patterns: higher Age (red dots on the right) and lower Barthel Index or SPO2_min (blue dots on the right) are the strongest predictors associated with a positive infection diagnosis.

Finally, Figure 9 presents the SHAP beeswarm plot, which illustrates the contribution of each feature to the model’s predictions across individual observations. Each point represents a data sample, colored according to the feature’s value (red = high, blue = low), with the horizontal position indicating the direction and magnitude of the impact. The plot highlights that for semantic features, higher frequencies (red) are strongly associated with increased predicted risk (points to the right). Furthermore, demographic factors such as Age show a clear correlation: higher values (red) are associated with a strong positive impact on the model output, reflecting the increased vulnerability of older populations, whereas lower values (blue) tend to reduce the predicted risk.

**Figure 10:**
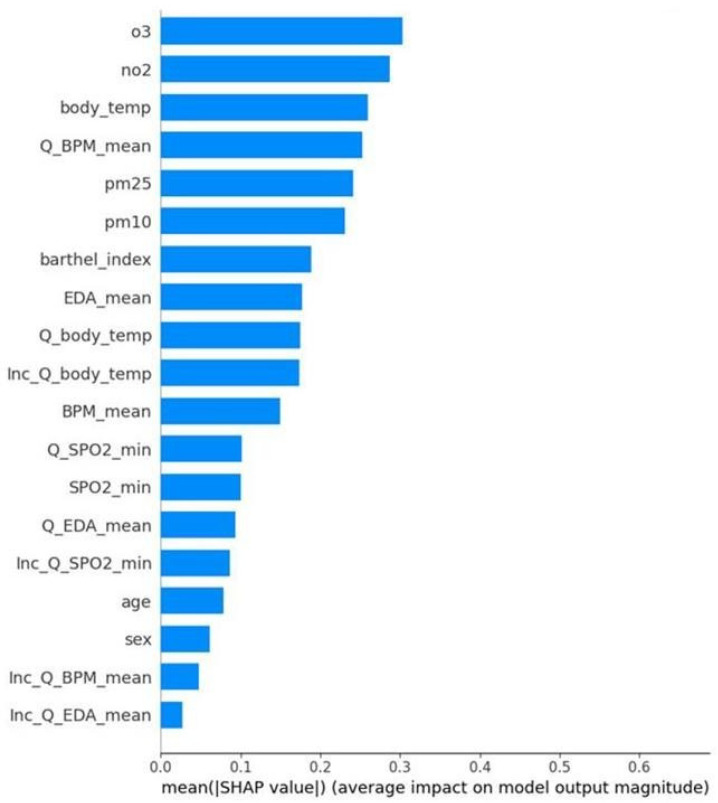
Feature importance ranking for the binary pollution-based model. The chart displays the mean absolute SHAP values, quantifying the global impact of each feature on the infection prediction. Air pollution variables, specifically Ozone (O_3_) and Nitrogen Dioxide (NO_2_), emerge as the top predictors. Notably, these gaseous pollutants show a higher predictive contribution than particulate matter (PM_25_, PM_10_) and rank slightly above critical physiological signals like body_temp.

Complementing the social analysis, the interpretation of the Air Pollution-based Model focuses on environmental determinants. Figure 10 shows the global importance of each variable in terms of mean absolute SHAP values for the binary classification task. The most influential features are pollution variables such as ozone (O_3_) and NO_2_. Physiological features such as Body Temperature and Q BPM mean also show relevant contributions, suggesting that both pollution and physiological factors are crucial for prediction.

**Figure 11:**
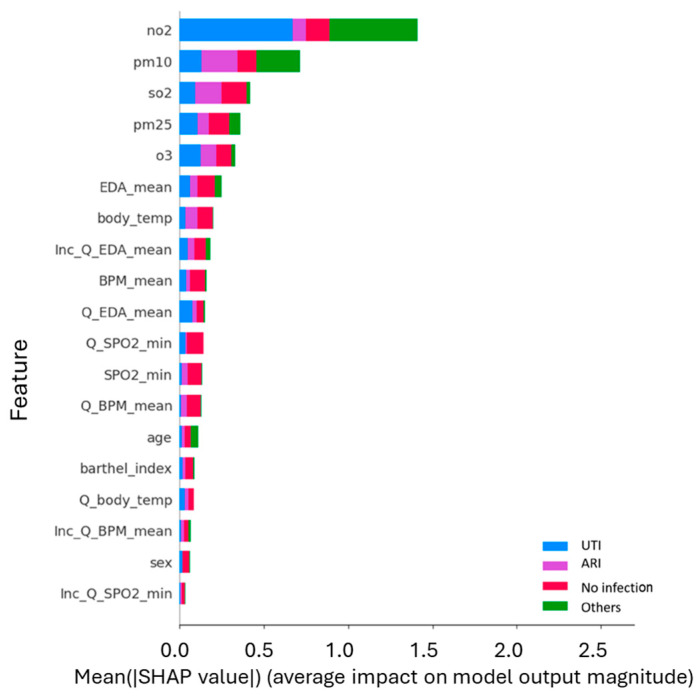
Feature contribution by class for the pollution-based multiclass model. The stacked bar chart ranks the variables by their mean absolute SHAP value, broken down by target class: UTI (Blue), ARI (Purple), No infection (Red), and Others (Green). Environmental variables occupy the top five positions, surpassing all physiological metrics. Nitrogen Dioxide (NO_2_) emerges as the strongest overall predictor, particularly influencing the detection of UTI and Others, whereas particulate matter (PM_10_) and Sulfur Dioxide (SO_2_) show a distinct contribution to identifying Respiratory Infections (ARI, purple segments).

In the multiclass scenario, Figure 11 presents the multiclass decomposition of feature importance, showing the average contribution of each feature to predictions across the four outcome classes. Air pollution factors, particularly NO_2_, PM_10_, and SO_2_, dominate the contribution for certain classes, while physiological features such as EDA mean and Body Temperature provide a more distributed influence across outcomes.

**Figure 12:**
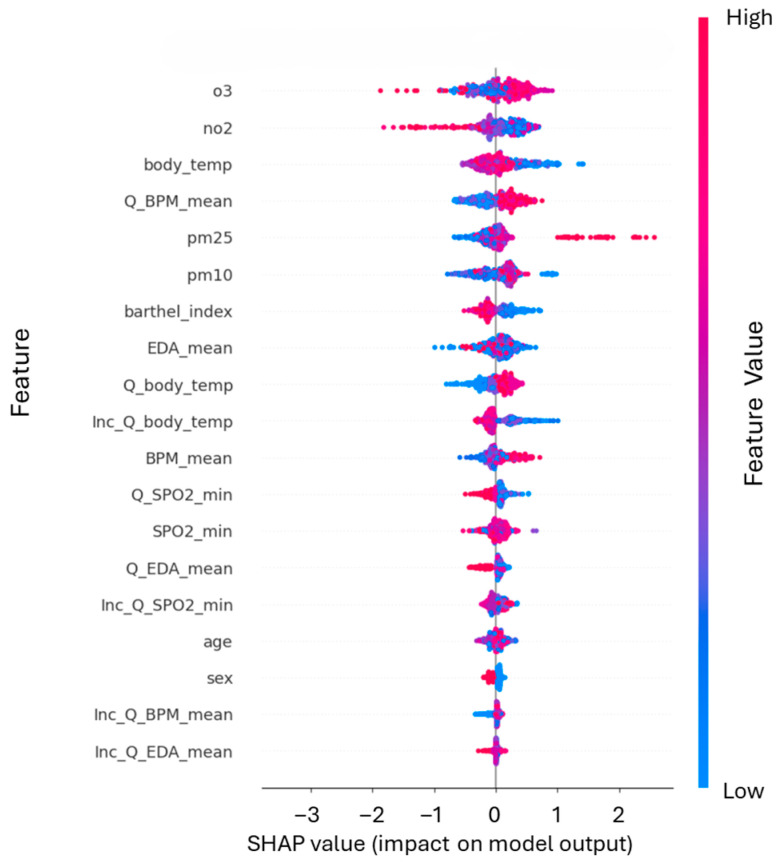
SHAP beeswarm plot illustrating the impact of air pollution variables on infection prediction. The plot combines feature importance (vertical ranking) with the direction of the effect. Each dot represents a sample, colored by its value (Red = High, Blue = Low). The distribution shows a clear pattern for the top features: high concentrations (red dots) of Ozone (O_3_) and Nitrogen Dioxide (NO_2_) are clustered on the positive side (right), indicating that elevated pollution levels significantly increase the model’s predicted probability of infection.

Lastly, the SHAP beeswarm plot in Figure 12 shows the distribution of SHAP values for individual predictions. Again, the color scale represents the feature value (red = high, blue = low). The plot reveals that high values of O_3_, NO_2_, and Body Temperature tend to drive the prediction toward higher risk, while lower values of physiological parameters such as SpO_2_ min push predictions toward lower risk. Together, these visualizations highlight the importance of integrating air pollution values with physiological data, demonstrating the model’s ability to capture complex interactions in the context of pollution-related risk prediction.

### 3.5. Summary of Main Findings

The experimental results indicate that integrating external data sources significantly enhances predictive performance compared to using physiological data alone, particularly in preventing the degradation of long-term forecasts. In the binary classification task, the Social Media Model emerged as a powerful forecasting tool, achieving a peak F1-Score of 0.97 with a 6-day lead time, whereas the Air Pollution Model provided robust stability for immediate detection (“nowcasting”). Crucially, in the multiclass scenario, external features resolved the “semantic gap” of the Basic Model—which failed to distinguish between pathologies (F1 < 0.50)—raising sensitivity for ARI and UTI to over 90% when using optimized feature sets. These findings validate the complementary nature of external and behavioral signals in overcoming the limitations of discontinuous clinical monitoring.

## 4. Discussion

The findings of this study provide a comprehensive insight into the integration and impact of external data sources—specifically, social media search trends and air pollution data—within machine learning models for the prediction of infections among nursing home residents. While physiological signs remain the standard for immediate clinical triage, our results demonstrate that they are insufficient for long-term forecasting or specific etiological diagnosis. The integration of air pollution and digital context fills this gap, transforming the model from a reactive detector into a proactive early warning system.

### 4.1. Comparison of Data Sources

A key contribution of this work is the identification of distinct temporal dynamics for each data source, suggesting a complementary rather than redundant relationship.

- Physiological Limits: The Basic Model proved effective for immediate detection but degraded rapidly as the prediction horizon extended. This suggests that vital signs act as indicators of current systemic stress rather than predictors of future risk, without immediate updates, their diagnostic relevance decays.
- Environmental Nowcasting: air pollution data consistently enhanced performance at short lags. The strong correlation found at Lag 0–2 supports the biological plausibility of environmental exposure—such as spikes in NO_2_—acting as immediate triggers for respiratory or systemic inflammation in vulnerable elderly populations.
- Social Forecasting: Contrary to the initial assumption that social media might add noise, our analysis revealed its critical role as a long-range sensor, peaking at a 6-day anticipation (F1 0.97). This aligns with the “digital epidemiology” hypothesis: community search patterns regarding symptoms precede clinical saturation, reflecting the behavioral latency between feeling the first symptoms and seeking medical attention.

### 4.2. Multiclass Classification Dynamics

The transition to multiclass classification (ARI vs. UTI) exposed the critical limitations of using physiological data in isolation. The Basic Model’s collapse (F1 < 0.48) illustrates the “Semantic Gap”: generic markers like fever or heart rate represent a systemic inflammatory response common to multiple infections, lacking the specificity required to distinguish the root cause.

In this context, external data acted as the decisive disambiguating factor. Social media features provided the semantic labels that vital signs lacked; by capturing specific keywords (e.g., “dysuria” vs. “cough”), the model could assign a specific etiology to a generic physiological anomaly.

Complementarily, pollution data improved ARI prediction compared to the baseline. This supports the hypothesis that air quality acts as a specific etiological driver for respiratory conditions, rather than just a generic stressor.

### 4.3. Impact of Feature Selection

Feature selection played a differentiated role depending on the task and data source. In binary classification, its effect was minimal across all settings. The feature set already contained sufficient discriminative power, and removing variables did not substantially alter results.

In contrast, multiclass classification showed that feature selection could either harm or help performance depending on the context. For the Basic Model (without external data), selection led to performance drops, likely due to the elimination of subtle but essential variables. However, with social media and especially with air pollution data, feature selection acted as a critical enhancer—filtering noise and emphasizing features most relevant to the target classes. This selective focus was particularly important in helping the model distinguish between clinically similar conditions. Although SHAP-based feature selection proved beneficial for certain multiclass scenarios, its overall impact across all settings was modest (2.6%). This is likely because averaging feature importance across all data tends to hide variables that are crucial only for small, specific classes. Essentially, by removing features that looked ‘unimportant’ on average, we may have accidentally discarded the unique signals needed to detect the rarer infections.

### 4.4. Model Explainability and Feature Contribution

SHAP analysis provided a critical layer of interpretability, validating that the predictive power of the models stems from genuine epidemiological signals rather than statistical artifacts. In the Social Media Model, the prominence of terms like cold, bronchitis, and flu (Figure 7) serves as a behavioral validation of the system, supporting the idea that it successfully detects the “digital footprint” of community health status. This interpretation is reinforced by the beeswarm plot (Figure 9), which reveals a directional consistency—higher frequencies of symptom terms increase infection probability—mirroring real-world transmission dynamics. Furthermore, the variation in feature contribution across classes in the multiclass setting (Figure 8) indicates that the model distinguishes specific linguistic patterns associated with different pathologies, rather than merely learning a generic “sick” label.

Complementarily, the Air Pollution Model (Figure 10 and Figure 11) supports the biological plausibility of the approach. High SHAP values for pollutants like NO_2_ and O_3_ align with established physiological mechanisms, capturing the external stressors that compromise the immune system. Unlike social media features, which reflect human behavior, these pollution variables—especially when lagged—capture the temporal delay between environmental exposure and the subsequent physiological reaction.

The impact of SHAP-based feature selection was particularly revealing for complex classes. By filtering out irrelevant variables, SHAP selection acted as a “signal amplifier”, isolating the subtle external triggers and specific keywords required to identify these less distinct pathologies. This suggests that for refined diagnosis, the quality and specificity of the signal are more critical than the sheer volume of data.

### 4.5. Handling Missing Data

An additional challenge addressed in this study was the high proportion of missing values in physiological variables, as noted in Table 3. Applying advanced imputation techniques proved essential, in line with previous studies [5,6]. This step ensured that models retained sufficient signal while minimizing bias, allowing for the integration of complete, reliable time series data.

### 4.6. Broader Implications

These results extend prior work from the SPIDEP project [3,4] reinforcing the potential of integrating multimodal data sources—including physiological, environmental, and behavioral signals—for early infection detection. Importantly, the success of the models depends not only on the inclusion of diverse data but also on strategic preprocessing, particularly imputation techniques for missing values, feature selection and temporal alignment. Carefully curated pollution metrics and filtered digital signals can substantially improve both the sensitivity and specificity of predictions.

Overall, this study supports the integration of environmental monitoring and digital epidemiology into predictive healthcare frameworks, offering practical value for anticipatory interventions in nursing home populations.

### 4.7. Limitations and Future Work

While the findings are robust, this study has limitations that must be acknowledged. First, the clinical dataset comes from a single nursing home, which may limit the direct generalizability of the findings to facilities with different demographics or protocols. Multi-center validation would be necessary to confirm the robustness of the models across broader populations.

Second, we acknowledge statistical limitations regarding the clustering of observations. Residents living in the same facility share environmental exposures and infection vectors, which challenges the assumption of independence underlying standard classifiers. However, our results suggest that this shared context is precisely what the environmental models capture. Future work could incorporate hierarchical or mixed-effects models to explicitly isolate facility-level clustering from individual physiological responses.

Third, despite the application of various imputation strategies, the high percentage of missing physiological data presents a challenge. While simple strategies like mean imputation provided sufficient stability for this retrospective analysis, the complexity of the missingness patterns limited the effectiveness of multivariate reconstruction methods (e.g., MICE, kNN). Consequently, real-time deployment would require more consistent data capture protocols to ensure reliability without heavy preprocessing.

Finally, the use of social media data introduces an inherent demographic bias, as the elderly population in nursing homes is not the primary user base of these platforms. The model relies on “proxy” signals (family members or general community discourse) rather than direct patient feedback. Future iterations could incorporate more direct behavioral monitoring or local sensor data within the facility to bridge this gap. Additionally, to address the operational feasibility of this solution in real-world scenarios, future studies will assess clinical adoption using the Technology Acceptance Model (TAM), ensuring the system aligns with the practical workflows and needs of healthcare professionals.

## 5. Conclusions

This study demonstrates the value and limitations of integrating complementary data sources—air pollution data and social media search trends—into predictive models for early infection detection in elderly populations residing in nursing homes. The binary classification task, for the prediction of generic infection, benefited significantly from social media data in terms of temporal anticipation, achieving high predictive scores (F1-score 0.97) with a 6-day lead time. In parallel, air pollution data provided substantial and consistent improvements across multiple performance metrics, especially recall and precision, highlighting its strong potential for immediate risk detection.

In the multiclass scenario, differentiating by infection type, the careful selection of features proved essential. Indiscriminate feature reduction negatively impacted performance in baseline models, whereas applying selection methods to additional data sources, particularly air pollution and social media, yielded significant class-specific improvements. These insights indicate that the strategic integration of environmental indicators and selectively chosen digital search data can notably enhance model accuracy and reliability.

Lastly, the robust analysis underscores the critical role of proper imputation techniques in managing missing data to maintain model performance, emphasizing their necessity for real-world healthcare analytics.

Collectively, these findings reinforce the importance of strategic data integration and rigorous methodological choices in developing effective predictive models for early infection detection, ultimately contributing to better healthcare outcomes for elderly residents in nursing homes. Finally, the significant social interest and the novelties of this research clear the path towards the potential implementation of this solution in real systems.

## Figures and Tables

**Table 1:** Description of the dataset made of the daily sampling of vital signs by the nurses and the diagnostics reported by the doctors, collected from the Cardenal Cisneros and Francisco de Vitoria nursing homes.

Population Metrics	Cardenal Cisneros	Francisco de Vitoria	Total
Residents	127	316	443
Participants	20	40	60
Infected Residents	7	33	40
Participation Rate (%)	16%	13%	14%
Medical Staff	4	14	18
Average Age	87.7	87.6	87.6
Start Date	24 March 2018	4 April 2018	24 March 2018
End Date	11 March 2019	11 March 2019	11 March 2019

**Table 2:** Characteristics of the diagnostic event data.

Category	Count
Acute Respiratory Infections	48
Urinary Tract Infections	54
Other Infections	50
Total Infections	152
Residents with Infections	43
Average Infections per Resident	3.53

**Table 3:** Summary of missing values for each vital sign.

Vital Sign	Expected Values	Actual Values	Missing Values	Missing Values (%)
Body temperature	21,002	4167	16,835	80.16
SpO_2_	21,002	4177	16,825	80.11
BPM	21,002	4178	16,824	80.11
EDA	21,002	4165	16,837	80.16

**Table 4:** Performance metrics for binary classification (Healthy vs. Infected). The results correspond to the Selected Feature Set (optimized via SHAP) using the Mean Imputation strategy.

Dataset	Class	With Feature Selection–Mean Imputation			
		Precision	Recall	Specificity	F1-Score
Basic	Healthy	0.81	0.73	-	0.77
	Infected	0.77	0.84	0.73	0.80
Social Media	Healthy	0.83	0.72	-	0.77
	Infected	0.76	0.86	0.72	0.81
Air Pollution	Healthy	0.85	0.77	-	0.81
	Infected	0.78	0.86	0.77	0.82

**Table 5:** Performance metrics for multiclass classification (Healthy vs. Acute Respiratory Infection (ARI) vs. Urinary Tract Infection (UTI) vs. Others). The results correspond to the Selected Feature Set (optimized via SHAP) using the Mean Imputation strategy.

Dataset	Class	With Feature Selection–Mean Imputation			
		Precision	Recall	Specificity	F1-Score
Basic	Healthy	0.37	0.52	0.59	0.43
	ARI	0.33	0.13	0.95	0.18
	UTI	0.45	0.53	0.66	0.49
	Other	0.22	0.10	0.92	0.13
Social Media	Healthy	0.81	0.90	0.90	0.85
	ARI	0.89	0.94	0.98	0.92
	UTI	0.88	0.97	0.93	0.92
	Other	0.87	0.51	0.98	0.63
Air Pollution	Healthy	0.81	0.94	0.89	0.87
	ARI	0.88	0.96	0.98	0.91
	UTI	0.90	0.96	0.94	0.93
	Other	0.86	0.49	0.98	0.61

## Data Availability

The data presented in this study are available on request from the corresponding authors due to confidentiality and privacy concerns regarding the participants (nursing home residents).

## Supplementary Materials

The following supporting information can be downloaded at: https://www.mdpi.com/article/10.3390/healthcare14020166/s1.
