# Supplementary material for: Predictive Models for Early Infection Detection in Nursing Home Residents: Evaluation of Imputation Techniques and Complementary Data Sources
Source: Healthcare (Basel). 2026 Jan 8;14(2):166. doi: 10.3390/healthcare14020166 (PMC12840934; doi:10.3390/healthcare14020166)
Supplement: Supplementary file 1 [file healthcare-14-00166-s001.zip › healthcare-4014250-supplementary.pdf]

healthcare

MDPI

Supplementary Material

As the analysis was conducted using search queries from Spain, the keywords used in the study were originally in Spanish. To ensure clarity for the international audience, Table S1 presents the Spanish terms alongside their English translations.

Table S1: Mapping between original Spanish search keywords and their English translations.

Spanish Keyword	English Translation
Fiebre	Fever
Resfriado	Cold
Catarro	Catarrh
Tos	Cough
Bronquitis	Bronchitis
Gripe	Flu
Antibióticos	Antibiotics
Infección	Infection
Paracetamol	Paracetamol
Disuria	Dysuria
Cistitis	Cystitis

Word Frequency and Temporal Trends

At the beginning of the analysis, a set of health-related search terms was selected, including Fever, Cold, Catarrh, Cough, Bronchitis, Flu, Antibiotics, Infection, Paracetamol, Dysuria, and Cystitis. The temporal variation of these terms throughout the study period is illustrated in Figure S1, which shows clear seasonal peaks, particularly during winter. These peaks were predominantly driven by respiratory symptoms, such as cold, catarrh, cough, bronchitis, and flu, which exhibited the most significant variations and were therefore selected for more detailed analysis due to their seasonal behavior and public health relevance.

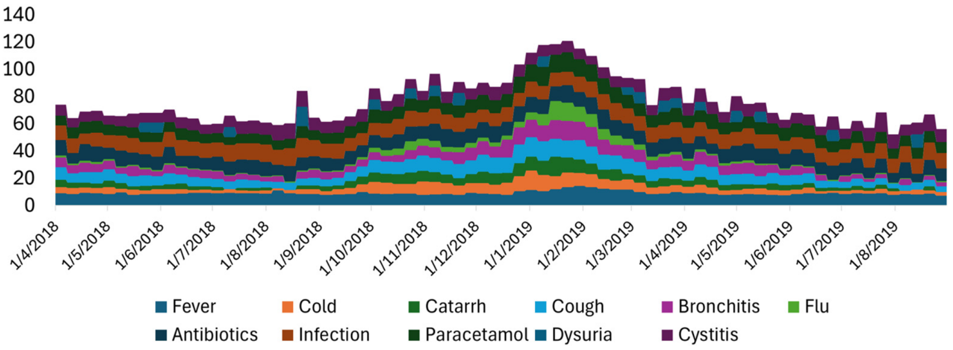

Figure S1: Seasonal evolution of health-related keyword searches on Google Trends. The stacked area chart aggregates the relative search volume for 11 specific symptoms and treatments (e.g., "Flu", "Fever", "Antibiotics") from April 2018 to August 2019. Note the significant surge in activity between December 2018 and February 2019, reflecting the expected seasonal peak of respiratory infections in the general population.

Further insights into respiratory symptom-related terms are provided in Figure S2, where synchronized peaks in flu and bronchitis searches during winter highlight their potential as early indicators of seasonal outbreaks. Searches for antibiotics and paracetamol followed similar patterns, reflecting increased public interest in symptom relief and treatment during these periods.

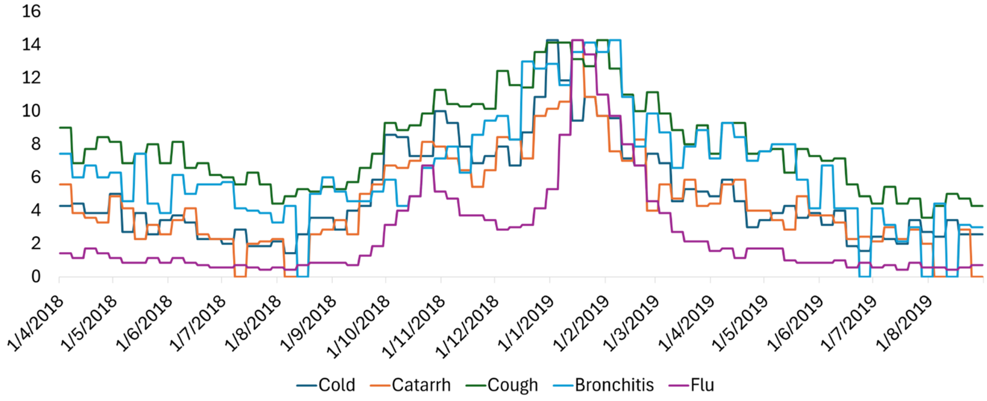

Figure S2. Comparative analysis of respiratory-related keyword search volume on Google Trends. This subset of data focuses on the relative search interest for five key respiratory symptoms (Cold, Catarrh, Cough, Bronchitis, Flu) from April 2018 to August 2019. The graph reveals high seasonal correlation, with search activity peaking sharply in January and February 2019. This validates the use of external environmental data to model the predictable seasonality of respiratory illnesses.

In contrast, Figure S3 examines the search behavior for urinary symptoms. While searches for cystitis remained relatively stable throughout the year, dysuria displayed intermittent peaks. These differing temporal patterns may reflect variations in underlying causes or influences, such as behavioral factors, seasonal exposure risks, or targeted public health campaigns.

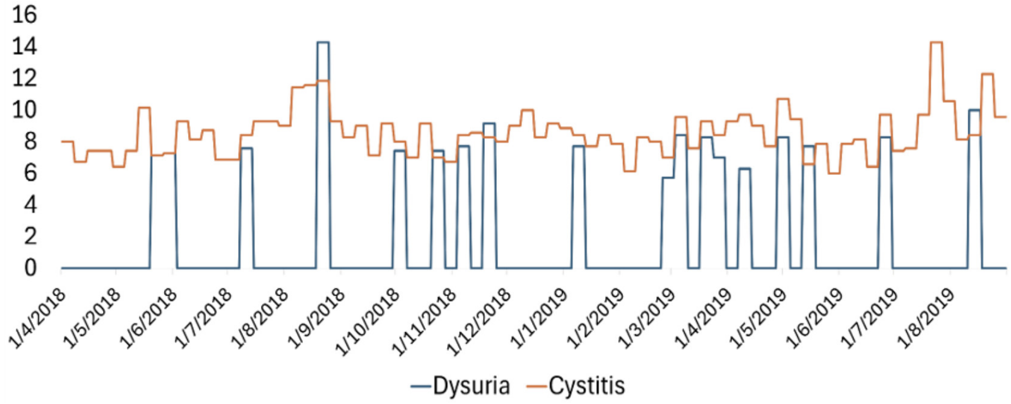

Figure S3. Comparative analysis of urinary symptom-related search trends. The graph displays the relative search volume for Dysuria (blue) and Cystitis (orange) over the same 16-month period used in the respiratory analysis. In stark contrast to respiratory symptoms, these urinary keywords exhibit a stochastic pattern with irregular spikes and no discernible seasonality. This confirms that urinary tract infections are independent of the environmental factors driving the winter outbreaks.

Taken together, these analyses reveal the significant influence of climatic and seasonal factors on the public's interest in health-related topics.
